# Supplementary material for: Understanding the health challenges of Amazonian riverine communities: A qualitative study on community perceptions amid climatic change
Source: PLoS One. 2025 Oct 10;20(10):e0333408. doi: 10.1371/journal.pone.0333408 (PMC12513581; doi:10.1371/journal.pone.0333408)
Supplement: S1 File — Consolidated criteria for reporting qualitative studies. (DOCX) [file pone.0333408.s001.docx]

**Consolidated criteria for reporting qualitative studies (COREQ): 32-item checklist - Understanding the Health Challenges of Amazonian Riverine Communities: A Qualitative Study on Community Perceptions Amid Climatic Change**

Developed from:

Tong A, Sainsbury P, Craig J. Consolidated criteria for reporting qualitative research (COREQ): a 32-item checklist for interviews and focus groups. *International Journal for Quality in Health Care*. 2007. Volume 19, Number 6: pp. 349 – 357

| **No. Item** | **Guide questions/description** | **Reported on Page #** |
| --- | --- | --- |
| **Domain 1: Research team and reﬂexivity** |  |  |
| *Personal Characteristics* |  |  |
| 1. Interviewer/facilitator | *Which author/s conducted the interview or focus group?*  APCS, EARTS and HSSG. | Data collection and analysis, 9. |
| 2. Credentials | *What were the researchers’ credentials?*  PhD and MSc. | Team reflexivity, 11. |
| 3. Occupation | *What was their occupation at the time of the study?*  University professors and qualitative researchers. | Title page. |
| 4. Gender | *Was the researcher male or female?*  Some researchers were male, others were female. | Team reflexivity, 11. |
| 5. Experience and training | *What experience or training did the researcher have?*  All with qualitative research training and with published articles in the area. | Team reflexivity, 11. |
| *Relationship with participants* |  |  |
| 6. Relationship established | *Was a relationship established prior to study commencement?*  None of the participants had an established relationship with an author prior to study commencement. | Team reflexivity, 11. |
| 7. Participant knowledge of the interviewer | *What did the participants know about the researcher? (e.g., personal goals, reasons for doing the research).*  We provided clear explanations of the study, objectives, procedures, and potential risks. | Ethical considerations, 6. |
| 8. Interviewer characteristics | *What characteristics were reported about the inter viewer/facilitator? e.g., bias, assumptions, reasons and interests in the research topic.*  No interviewer-related biases were identified. | Team reflexivity, 11. |
| **Domain 2: study design** |  |  |
| *Theoretical framework* |  |  |
| 9. Methodological orientation and Theory | *What methodological orientation was stated to underpin the study? e.g., grounded theory, discourse analysis, ethnography, phenomenology, content analysis.*  Thematic analysis. | Data analysis, 10. |
| *Participant selection* |  |  |
| 10. Sampling | *How were participants selected? e.g., purposive, convenience, consecutive, snowball.*  Purposive sample. | Study area and population, 8. |
| 11. Method of approach | *How were participants approached? e.g., face-to-face, telephone, mail, email.*  We did a face-to-face interview. | Data collection, 9. |
| 12. Sample size | *How many participants were in the study?*  32 individuals | Results, 11. |
| 13. Non-participation | *How many people refused to participate or dropped out? Reasons?*  No one has given up on participating in the project. | N/A |
| *Setting* |  |  |
| 14. Setting of data collection | *Where was the data collected? e.g., home, clinic, workplace.*  The IDIs and FGDs were carried out at their house, school or church community. | Data collection, 10. |
| 15. Presence of non-participants | *Was anyone else present besides the participants and researchers?*  No, only the interviewer, the observer and the participant were in the room during the interview. | Data collection, 10. |
| 16. Description of sample | *What are the important characteristics of the sample? e.g. demographic data, date*  We putted this information in the results section. | Results, 11. |
| *Data collection* |  |  |
| 17. Interview guide | *Were questions, prompts, guides provided by the authors? Was it pilot tested?*  The questions were developed by Qualitative researchers and previously tested and validated by the researchers. | Data collection,9-10. |
| 18. Repeat interviews | *Were repeat inter views carried out? If yes, how many?*  N/A | N/A |
| 19. Audio/visual recording | *Did the research use audio or visual recording to collect the data?*  The interviews were recorded in audio and transcribed without personal identifiers, so that the database could be anonymized | Data collection, 10. |
| 20. Field notes | *Were ﬁeld notes made during and/or after the inter view or focus group?*  Fieldnotes were made in notebook. | Data collection, 10. |
| 21. Duration | *What was the duration of the interviews or focus group?*  The interviews lasted an average of 40 and 60 minutes. | Data collection, 10. |
| 22. Data saturation | *Was data saturation discussed?*  The number of interviews was determined by the principle of theoretical saturation where IDIs are carried out until a clear pattern appears and subsequent groups do not produce new information. | Study area and population, 8. |
| 23. Transcripts returned | *Were transcripts returned to participants for comment and/or correction?*  No. | N/A |
| **Domain 3: analysis and ﬁndings** |  |  |
| *Data analysis* |  |  |
| 24. Number of data coders | *How many data coders coded the data?*  Two researchers developed a codebook and performed line-by-line coding. | Data analysis, 10. |
| 25. Description of the coding tree | *Did authors provide a description of the coding tree?*  No. | N/A |
| 26. Derivation of themes | *Were themes identiﬁed in advance or* *derived from the data?*  The analysis of the interviews and the field notes allowed us to identify the two themes. | Data analysis, 10. |
| 27. Software | *What software, if applicable, was used to manage the data?*  The recordings of the IDIs and FDGs were transcribed and inserted in the MAXQDA 20 program. | Data analysis, 10. |
| 28. Participant checking | *Did participants provide feedback on the ﬁndings?*  No. | N/A |
| *Reporting* |  |  |
| 29. Quotations presented | *Were participant quotations presented to illustrate the themes/ﬁndings? Was each quotation identiﬁed? e.g. participant number*  Yes, quotations were presented to illustrate the themes/findings, and each quotation was identified with a participant number. | Results, 10-19. |
| 30. Data and ﬁndings consistent | *Was there consistency between the data presented and the ﬁndings?*  Yes, there was consistency between the data presented and the findings. | Discussion, 19-24. |
| 31. Clarity of major themes | *Were major themes clearly presented in the ﬁndings?*  Yes, the themes were clearly presented in the Results section using specific sections regarding each theme. | Results, 10-19. |
| 32. Clarity of minor themes | *Is there a description of diverse cases or discussion of minor themes?*  No, minor themes were not discussed. | N/A |
