# Supplementary material for: Understanding the health challenges of Amazonian riverine communities: A qualitative study on community perceptions amid climatic change
Source: PLoS One. 2025 Oct 10;20(10):e0333408. doi: 10.1371/journal.pone.0333408 (PMC12513581; doi:10.1371/journal.pone.0333408)
Supplement: S2 File — Inclusivity in global research. (DOCX) [file pone.0333408.s002.docx]

Inclusivity in global research

PLOS’ policy on inclusivity in global research aims to improve transparency in the reporting of research performed outside of researchers’ own country or community and ensures that PLOS publications reporting global research adhere to high standards for research ethics and authorship. Authors of relevant research articles may be asked to complete the questionnaire below, which outlines ethical, cultural, and scientific considerations specific to inclusivity in global research. This questionnaire may be requested when researchers have travelled to a different country to conduct research, if research uses samples collected in another country, research with Indigenous populations or their lands, or if research is on cultural artefacts. Researchers travelling to another country solely to use laboratory equipment will not normally be required to complete the questionnaire. However, the questionnaire can be requested at the journal’s discretion for any submission – if you have been requested to complete this questionnaire by the PLOS journal you submitted to, please do so.

Please complete the questionnaire below and include this as a Supporting Information file with your manuscript. Note that if your paper is accepted for publication, this checklist will be published with your article in the supporting information files. Please ensure that you reference the checklist in the main body of your manuscript. We suggest adding a subsection ‘Inclusivity in global research’ to your Methods section and adding the following sentence: “Additional information regarding the ethical, cultural, and scientific considerations specific to inclusivity in global research is included in the Supporting Information (SX Checklist)”

The questions have been designed to be applicable to a wide range of study types, and there are subsections for both human subjects research and non-human subjects research. If any of the questions are not relevant to your research please mark them as “N/A” as appropriate.

**Ethical considerations, permits and authorship**

*This section is applicable to all research types.*

Provide details as to who granted permissions and/or consent for the study to take place in the Methods section of your manuscript. This should include the names of **all** ethics boards, governmental organizations, community leaders or other bodies that provided approval for the study. If individuals provided approval refer to these people by their role or title but do not list their name(s).

Reported on page number: 6 and 7.

If there were any deviations from the study protocol after approval was obtained please provide details of these changes in the Methods section of your manuscript.

Reported on page number: 6 and 7.

Did this study involve local collaborators that are residents of the country where the research was conducted or members of the community studied? If you do not have any authors from said communities, please provide an explanation for this below.

The study was conducted by Brazilian researchers affiliated with Brazilian institutions, specializing in tropical diseases and qualitative methods. While the research actively engaged members of the riverine community as participants (interviewees and focus group), authorship was limited to external researchers. Although this approach is common in institutionally led qualitative studies, it underscores the need for future research to incorporate local co-authors to enhance representativeness and ensure community ownership of the findings.

Everyone listed as an author should meet PLOS’ criteria for authorship and all individuals who meet these criteria should be included in the author byline, rather than the acknowledgements. For further information please see the journal’s Authorship Policy.

**Human subjects research (e.g. health research, medical research, cross-cultural psychology)**

Did you obtain written informed consent from a representative of the local community or region before the research took place? How did you establish who speaks for the community? Details of written informed consent obtained from study participants should be reported separately in the Methods section of your manuscript.

The study engaged local community representatives through a formal pre-research consultation meeting with key stakeholders, including the community association president, school principal, teachers, and religious leaders. During this meeting, the research team presented and discussed: Research aims - The health challenges related to climatic changes affecting riverine populations Methodology - The planned qualitative approach using interviews and focus groups Anticipated outcomes - Potential benefits for healthcare access improvement This consultation resulted in verbal approval aligned with local decision-making customs, which was documented in research field notes. While the manuscript emphasizes individual informed consent procedures, this community-level engagement ensured cultural appropriateness and addressed the principle of community autonomy in research involving traditional populations.

Written informed consent was obtained from all adult study participants, as detailed in the "Ethical Statement" section of the manuscript. For participants under 18 years of age, consent was secured through formal authorization from parents/legal guardians, supplemented by verbal assent from the adolescents. Beyond individual protocols, a prior community consultation process was conducted involving a formal meeting with the local association president, school principal, teachers, and religious leaders, during which the project was presented and verbally approved - a practice aligned with local customs that was properly documented in field notes by the research team. This approach ensured not only institutional ethical compliance but also recognition of traditional decision-making mechanisms within the studied riverine community.

How did members of the local community provide input on the aims of the research investigation, its methodology, and its anticipated outcome(s)?

When engaging with the local community, how did you ensure that the informed consent documents and other materials could be understood by local stakeholders?

The study ensured comprehension of informed consent materials by the riverine community through multiple context-adapted strategies. Documents were prepared in Portuguese using accessible language and supplemented with detailed verbal explanations, as described in the article's ethics section. For participants with limited formal education, the team employed concrete examples and colloquial language, while for adolescents (12-17 years old), an age-appropriate approach was adopted, obtaining parental consent complemented by verbal assent. The process included continuous oral revalidation when new topics emerged during research, as documented in field records. Prior approval obtained from community leaders (association president, school principal, teachers, and religious figures) ensured culturally appropriate communication.

Will the findings of the research be made available in an understandable format to stakeholders in the community where the study was conducted (e.g. via a presentation, summary report, copies of publications, etc.)? Please provide details of how this will be achieved.

Yes, the findings will be shared with the riverine community through community assemblies and simplified Portuguese-language reports containing the main results and recommendations.

**Non-human subjects research using specimens/ animals collected as part of the study, or those housed in archival collections. Examples include archaeology, paleontology, botany and zoology.**

Did the permission you obtained from a local authority to perform the study include an agreement on access to outputs and benefit sharing? This may include procedures to enable fair distribution of the benefits and resources arising from the research performed. Please include any details of Prior Informed Consent and Benefit Sharing Agreements obtained. These may be required by field-specific regulations, for example the Convention on Biological Diversity (CBD) and the associated Nagoya Protocol.

N/A.

If the material used in your study was imported, please A) provide the year it was imported and B) indicate whether permits were obtained to import/export the materials used, C) provide details of any permits obtained. If this information is not available, please indicate this.

N/A.

If you used archival specimens, please state how the material used in your study was acquired by the institute it is held in and provide details of any permits obtained for the original excavations/ sample collection. If this information is not available, please indicate this.

N/A.

How was the potential cultural significance of the materials collected in your study to local communities considered in your research design? Were Indigenous peoples and/or local researchers and institutions involved with archaeological excavations / collection of specimens? If so, please provide a description of their involvement.

N/A.

If your manuscript includes photographs of human remains please indicate whether authors obtained permission from descendants or affiliated cultural communities to do so.

N/A.
