# Supplementary material for: Understanding the health challenges of Amazonian riverine communities: A qualitative study on community perceptions amid climatic change
Source: PLoS One. 2025 Oct 10;20(10):e0333408. doi: 10.1371/journal.pone.0333408 (PMC12513581; doi:10.1371/journal.pone.0333408)
Supplement: S3 File — (PDF) [file pone.0333408.s003.pdf]

Codebook: Understanding the Health Challenges of Amazonian Riverine Communities: A Qualitative Study on Community Perceptions Amid Climatic Change

Riverine and climate change_Alicia.mx20

16/01/2025

Codes and Subcodes	Nº of segments coded
**1 Health and Amazonian Seasonality**	**418**
1.1 River as a Determinant of Lifestyle	53
1.2. Health Problems During the Amazonian Summer	103
1.3 Problems During the Amazonian Winter	141
1.4 River as a Source of Food and Water	48
1.5 River Pollution and Its Impact on Health	73
**2. Challenges in Accessing Healthcare**	**337**
2.1 Searching for Care Facilities	77
2.2 Barriers to Accessing Healthcare	131
2.3 Feeling Abandoned	76
2.4 Deaths and Births in the Search for Care	53
**Total of segments coded**	**755**
