# Supplementary material for: Understanding the health challenges of Amazonian riverine communities: A qualitative study on community perceptions amid climatic change
Source: PLoS One. 2025 Oct 10;20(10):e0333408. doi: 10.1371/journal.pone.0333408 (PMC12513581; doi:10.1371/journal.pone.0333408)
Supplement: Attachment — Submitted filename: Response to Reviewers.pdf [file pone.0333408.s006.pdf]

Response to Reviewers

Review Comments to the Author

Please use the space provided to explain your answers to the questions above. You may also include additional comments for the author, including concerns about dual publication, research ethics, or publication ethics. (Please upload your review as an attachment if it exceeds 20,000 characters)

Reviewer #1: Congratulations again on your work. It's very stimulating to read works like this. I have just one observation to make. The first time the acronyms referring to the researchers' names appear is in lines 218-219. However, the explanation for these acronyms only appears in the "research team and reflexivity" section, on line 245. I suggest correcting this to facilitate the reader's understanding. Thank you for your dedication in following my considerations.

Answer: We sincerely appreciate your thoughtful feedback and are grateful for your recognition of our work. To improve reader comprehension, we have moved the explanation of author acronyms from the "Research Team and Reflexivity" section to their first appearance in the Methods section (Lines 212-215).

Reviewer #2: All prior recommendations were addressed satisfactorily.

General Notes

Your ethical documentation (Lines 135-139) now robustly addresses consent protocols, and the COREQ adherence strengthens methodological transparency. The tri-border focus provides uniquely valuable insights for climate-vulnerable regions globally. These final refinements will elevate an already compelling study:

1. Ancestral Futures Integration (Lines 572-581)

Please ground the "ancestral futures" discussion in concrete participant data. After the phrase "holistic vision requires" in Line 534, insert: "For example, participants described using moringa seed filtration to purify smoke-contaminated rainwater during droughts practice elders identified as historically effective. Integrating such techniques into mobile clinic protocols could immediately reduce gastrointestinal illnesses while honoring traditional knowledge."

Answer: The information was included (line 540-551).

2. Clarity in Cross-National Context (Line 444)

The reference to subsistence farming impacts [26] lacks geographic specificity, creating ambiguity about whether findings derive from your study area or comparative research. Please replace "subsistence farming[26]" with "subsistence farming in Peru [26]" to clarify the international comparison and prevent misinterpretation of regional evidence.

Answer: The information was added (line 438).

3. Abstract Structure (Lines 54-57)

The first sentence in the conclusions section ("Tailored healthcare solutions such as sustainable infrastructure, including telemedicine platforms, mobile clinics, and resilient transportation networks.") is grammatically incomplete due to the absence of a main verb. Recommended Revision: "Tailored healthcare solutions, including telemedicine platforms, mobile clinics, and resilient transportation network, are urgently needed. Investments in communication infrastructure and emergency air transportation are critical as riverine navigation becomes increasingly unreliable."
*Eliminates ambiguity and strengthens the abstract's persuasiveness for policymakers.

Answer: The information was changed (Line 54-58).
